# Health-Related Quality of Life and Health Resource Utilization in Patients with Primary Immunodeficiency Disease Prior to and Following 12 Months of Immunoglobulin G Treatment

**DOI:** 10.1007/s10875-016-0279-0

**Published:** 2016-04-18

**Authors:** John Routes, Beatriz Tavares Costa-Carvalho, Bodo Grimbacher, Kenneth Paris, Hans D. Ochs, Alexandra Filipovich, Mary Hintermeyer, Karina Mescouto de Melo, Sarita Workman, Diane Ito, Xiaolan Ye, Patrick Bonnet, Josephine Li-McLeod

**Affiliations:** Medical College of Wisconsin and the Children’s Research Institute, Milwaukee, WI USA; Section of Allergy and Clinical Immunology, Department of Pediatrics, Medical College of Wisconsin, 9000 W. Wisconsin Ave., Milwaukee, WI 53226-4874 USA; Universidade Federal de Sao Paulo, Sao Paulo, Brazil; Institute of Immunity & Transplantation, Royal Free Hospital, University College London, London, UK; Center for Chronic Immunodeficiency, University Hospital Freiburg, Freiburg im Breisgau, Germany; LSU Health Sciences Center, Children’s Hospital New Orleans, New Orleans, LA USA; Seattle Children’s Research Institute and University of Washington, Seattle, WA USA; Cincinnati Children’s, Cincinnati, OH USA; The Royal Free London NHS Foundation Trust, London, UK; Baxalta US, Inc, Cambridge, MA USA; Baxalta US, Inc, Bannockburn, IL USA

**Keywords:** Primary immunodeficiency, antibody deficiency, health-related quality of life, health resource utilization, short form health survey (SF-36), pediatric quality of life inventory (PedsQL)

## Abstract

**Purpose:**

Health-related quality of life (HRQOL) has not been examined in patients with predominant antibody deficiency both pre- and post-immunoglobulin G (IgG) treatment initiation. HRQOL and health resource utilization (HRU) were assessed in newly diagnosed patients with primary immunodeficiency disease (PIDD) pre- and 12 months post-IgG treatment initiation.

**Methods:**

Adults (age ≥18 years) completed the 36-item Short Form Health Survey, version 2; pediatric patients (PP)/caregivers completed the Pediatric Quality of Life Inventory (PedsQL). Scores were compared with normative data from the US general population (GP) and patients with other chronic conditions (OCC).

**Results:**

Seventeen adult patients (APs), 8 PPs, and 8 caregivers completed baseline assessments. APs had significantly lower baseline mean physical component summary scores versus GP (37.4 vs 50.5, *p* < 0.01) adults with chronic back pain (44.1, *p* < 0.05) or cancer (44.4, *p* < 0.05) and lower mental component summary scores versus GP (41.6 vs 49.2, *p* < 0.05). PPs had lower PedsQL total (63.1 vs 82.7), physical summary (64.5 vs 84.5), and psychosocial summary (62.5 vs 81.7) scores versus GP. Post-IgG treatment, 14 APs, 6 PPs, and 8 caregivers completed assessments. Hospital admissions (0.2 versus 1.8, *p* < 0.01), serious infections (3.3 versus 10.9, *p* < 0.01) and antibiotic prescriptions (3.0 versus 7.1; *p* < 0.01) decreased significantly overall. While APs reported significant improvement in role-physical (*p* = 0.01), general health (*p* < 0.01), and social functioning (*p* = 0.02) and caregivers in vitality (*p* < 0.01), PPs did not.

**Conclusions:**

Pre-IgG treatment, patients with PIDD experienced diminished HRQOL versus GP and patients with OCC; post-treatment, HRU decreased and certain HRQOL aspects improved for APs and caregivers.

## Introduction

Primary immunodeficiency diseases (PIDDs), a heterogeneous group of disorders that include antibody deficiencies [1–4], are characterized by increased susceptibility to infections [1]. Patients with predominant antibody deficiencies can present in infancy or adulthood [5] and are particularly vulnerable to bacterial infections that may result in chronic lung disease, bronchiectasis, and gastrointestinal symptoms [2, 4, 6–8]. Affected patients experience a range of symptoms that require lifelong treatment. Antibody replacement with immunoglobulin G (IgG) is the most commonly utilized prophylactic/therapeutic approach for the management of antibody deficiency disorders, and IgG administered intravenously or subcutaneously has been shown to safely and effectively reduce the frequency and severity of infections in these patients [9–16].

PIDD imposes a significant disease burden on patients, including limitations in work, play, or normal physical activity [17, 18]. Quality of life, including health-related quality of life (HRQOL), in patients with PIDD can be significantly affected by delays in diagnosis [1, 19–24]. Currently, these delays are considerable, as data from a patient survey in the United States indicated that the average time from the onset of symptoms associated with PIDD to the time of diagnosis is 12.4 years [17], similar to or longer than published reports of average delays of 12.5 years [25], 4.7 years [26], and 4.4 years [27] from other countries. Furthermore, following treatment initiation, patients have also reported lower HRQOL compared with the general population [18, 23, 28–32]. However, details of the HRQOL burden patients with PIDD experience both before diagnosis and after IgG treatment initiation are lacking.

In this study we investigated the burden of disease among patients and caregivers of pediatric patients newly diagnosed with PIDD, focusing on patients with impaired antibody production, and assessed the impact of IgG treatment on patient quality of life. Our primary goal was to gain insight into the effect of infections and impairments on HRQOL prior to and after initiation of treatment with IgG in patients with predominantly antibody deficiencies. We present comparisons of HRQOL between patients with PIDD and age- and sex-matched patients with other chronic conditions and the general US population. In addition, we present an assessment of healthcare utilization in the 6-month period preceding the start of and 12-months following treatment initiation with IgG.

## Methods

### Study Design

A 12-month, prospective, uncontrolled, open-label, observational study was initiated in 2010 to assess the burden of disease among patients with newly diagnosed predominant antibody deficiencies and, following initiation with IgG treatment, the impact of treatment on quality of life and health resource utilization. Patients were recruited from six Jeffrey Modell Foundation treatment centers in the United States, Brazil, and the United Kingdom. Participating study sites offered a financial incentive to study participants in the form of (1) two $25 checks, (2) up to $50 for reimbursement of transportation costs, or (3) no financial incentive, depending on the local regulatory requirements. The appropriate institutional review board or ethics committee at each site or country approved the study protocol.

### Participants

The study focused on patients with predominant antibody deficiencies in the absence of significant T-cell defects. Eligible patients were recently diagnosed with or suspected of having common variable immunodeficiency (CVID), XLA or autosomal recessive agammaglobulinemia, or specific antibody deficiency. Diagnoses of PIDD that require treatment beyond regular IgG replacement (eg, severe combined immunodeficiency and Wiskott-Aldrich Syndrome) were excluded. All diagnoses of PIDD were confirmed by laboratory testing and all patients were symptomatic (ie, had recurrent infections) and deemed suitable for IgG treatment. Both pediatric (age <18 years) and adult (age ≥18 years) patients who qualified for IgG replacement but had not yet started therapy were included. Patients with a life expectancy of <1 year, dementia or mental incapacity, or with comorbidities or other conditions that would interfere with the data interpretation were excluded. Patients who were currently receiving IgG or had received IgG in the recent past were also not eligible for the study. Patients or their caregivers provided written informed consent and were enrolled in the study from September 2010 to December 2012. IgG treatment regimen and dosing intervals were per the discretion of the treating physician.

### Data Collection

Patients were assessed at two time points. Baseline assessment occurred after confirmation of the diagnosis and during the 2 weeks before first IgG infusion or on the first day of treatment. The follow-up assessment took place 12 months after initiation of IgG treatment (±1 month), with the last patient follow-up completed in December 2013. At each assessment, serious infections, or other acute or chronic infections (eg, acute chronic sinusitis; bronchitis; bacterial pneumonia; bacteremia or sepsis; osteomyelitis or septic arthritis; visceral abscess; bacterial meningitis; and ear infection) were captured, and patients completed self-administered questionnaires to evaluate quality of life and health resource utilization. Pediatric patients aged 8–17 years completed the questionnaire with the help of their caregiver (parent/guardian). Caregivers completed the questionnaire on behalf of patients aged <8 years. Collected data were entered into an electronic database for analysis.

Adult patients and caregivers of pediatric patients completed the 36-item Short Form Health Survey, version 2 (SF-36v2) [33, 34]. The SF-36v2 survey is a validated and frequently used [35, 36] measure of HRQOL that evaluates eight domains related to functional health status and emotional well-being. These eight domains can be categorized into higher-order clusters of physical health (physical function, role—physical, bodily pain, general health) and mental health (vitality, social function, role – emotional, and mental health).

Pediatric patients (age <18 years) were evaluated with the Pediatric Quality of Life Inventory (PedsQL) measurement instrument [37]. The PedsQL survey is a validated, reliable [38] measure of HRQOL specifically for the pediatric population that evaluates physical, emotional, social, and school-related parameters.

A health resource utilization questionnaire was also administered at baseline to collect information on hospitalizations, emergency room visits, antibiotic use and days missed from work or school in the 6 months prior to enrollment. Patients were also given a diary for recording healthcare utilization during the 12-month study period.

### Statistical Analysis

The primary endpoint for the analysis was the baseline and the 12-month follow up quality-of-life (QOL) score. Secondary endpoints included occurrence of hospitalization, emergency room visits, office visits for PIDD-related events, and use of medications, such as antibiotics for these time points. Descriptive statistical analyses were carried out for all primary and secondary endpoint parameters.

Using the normative data available for the SF-36v2, comparative samples were age- and sex-matched using separate least squares multiple regression models for each QOL scale and summary measure. While the SF-36v2 normative database is based on US respondents only, research has shown that the US-based norms are valid and appropriate for interpreting scores of respondents from other countries [39].

The SF-36v2 normative database allowed for comparisons to a general US population as well as subgroups within that population with the following chronic conditions: rheumatoid arthritis, chronic obstructive pulmonary disease (COPD), congestive heart failure (CHF), cancer, and chronic back pain. A univariate analysis of variance model was used to test differences between the SF-36v2 scores for patients with PIDD and patients from the comparative groups. The PedsQL has published normative data from healthy and chronic conditions, including asthma, cancer, diabetes, and rheumatology for comparison purposes [37]. Because the normative data for the PedsQL are in published form only, 95 % confidence intervals were used to assess differences between scores for patients with PIDD and patients from the comparative groups.

A power calculation to determine the appropriate sample size for this study was not conducted due to the lack of historical results to estimate the potential effect size for the HRQOL endpoints and due to the exploratory nature of the study without a formal hypothesis formulated or tested. The study aimed to include 30 or more patients with PIDD; due to the rarity of PIDD and the challenge of finding newly diagnosed patients naïve to IG therapy, such a number was considered to be reasonably attainable, while adding valuable information to conduct an exploratory analysis of the stated endpoints.

### Determination of Clinically Relevant Differences

Changes in SF-36 scores prior to and following IgG treatment initiation were compared with known minimally important differences (MID) for each category [40]. Changes that met or exceeded the MID threshold were considered to be clinically meaningful.

## Results

### Patient Characteristics

A total of 31 patients (11 each from the United States and Brazil, and 9 from the United Kingdom) were enrolled in the study. The enrolled population included 21 (66.7 %) adult patients and 10 (33.3 %) pediatric patients. Six patients discontinued early due to treatment noncompliance (1 adult), home relocation (1 pediatric), and unknown reasons (3 adults and 1 pediatric). Baseline assessments were completed by 17 adult patients, 8 pediatric patients, and 8 caregivers, while 12-month assessments were completed by 14 adults, 6 pediatric patients, and 8 caregivers. Missing data from 2 of the pediatric patients precluded their inclusion in the follow-up analyses.

Baseline characteristics for the 25 patients and 8 caregivers included in the analysis are summarized in Table 1. The mean age was 47.7 years (standard deviation [SD] = 12.9) for adult patients and 5.1 years (SD = 4.8) for pediatric patients. CVID was the most frequently occurring diagnosis amongst the adults (14/21, 66.6 %), while hypogammaglobulinemia was the most common diagnosis among the pediatric patients (4/8, 50 %).

**Table 1:** Patient characteristics

Characteristic	Patients		Caregivers (Mothers) (*N* = 8)
	Adult (age ≥18 years) (*N* = 17)	Pediatric (age <18 years) (*N* = 8)	
Age, years			
Mean (SD)	47.1 (13.2)	5.5 (5.4)	32.8 (8.6)
Min-max	24–67	0–17	23–47
Sex, n (%)			
Male	6 (35.3)	5 (62.5)	0 (0)
Female	11 (64.7)	3 (37.5)	8 (100)
Diagnosis, n (%)			
CVID	11 (64.7)	2 (25)	N/A
Other^a^	6 (35.3)	6 (75)	N/A
Agammaglobulinemia	1 (5.9)	0 (0)	N/A
Hypogammaglobulinemia	2 (11.7)	4 (50)	N/A
Specific antibody deficiency	3 (17.6)	2 (25)	N/A
Working status, n (%)			
Work full-time (≥35 h/week)	9 (52.9)	N/A	2 (25.0)
Work part-time (<35 h/week)	2 (11.8)	N/A	2 (12.5)
Stay-at-home parent	0 (0)	N/A	1 (12.5)
Disabled	1 (5.9)	N/A	1 (12.5)
Unemployed	0 (0)	N/A	3 (37.5)
Retired	4 (23.5)	N/A	0 (0)
Other	1 (5.9)	N/A	0 (0)
Marital status, n (%)			
Single	4 (23.5)	N/A	1 (12.5)
Married	103 (58.8)	N/A	4 (50.0)
Divorced	1 (5.9)	N/A	3 (37.5)
Other	2 (11.8)	N/A	0 (0)
Race			
White or Caucasion	17 (100.0)	7 (87.5)	N/A
African-American	0 (0)	1 (12.5)	N/A
Location of infusions			
Home	2 (11.8)	0 (0)	N/A
Infusion center	15 (88.2)	8 (100)	N/A

^a^Patients in this category were all deemed suitable candidates for the current study and for IgG replacement treatment

*CVID* common variable immunodeficiency (CVID), *IgG* immunoglobulin G), *max* maximum), *min* minimum), *N*/*A* not applicable), *SD* standard deviation

### QOL Assessment in Adult Patients

**Fig. 1:**
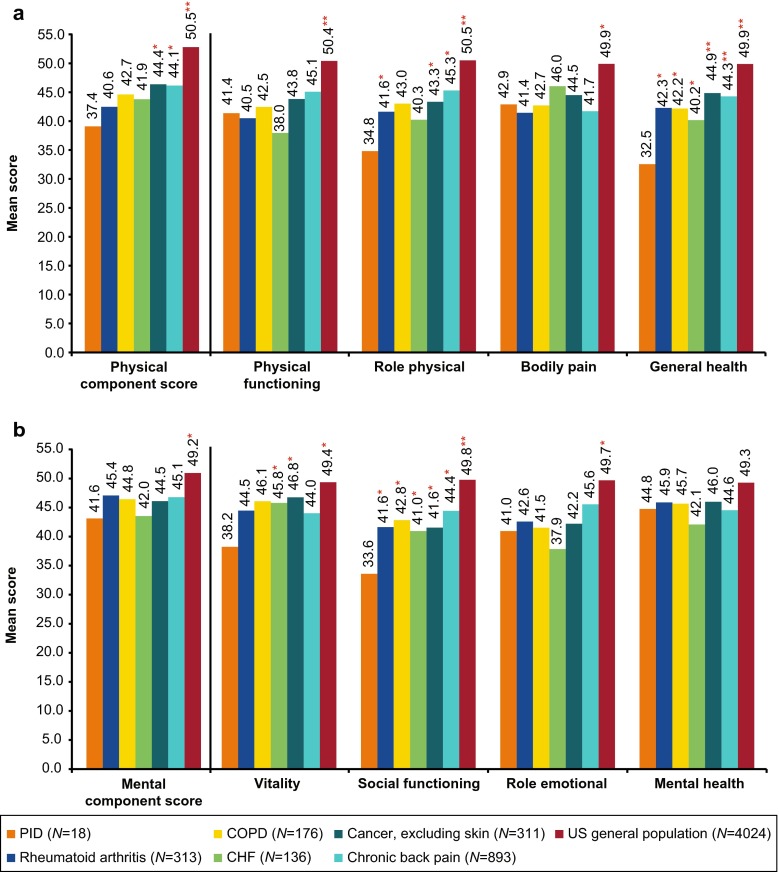
SF-36 physical (**a**) and mental (**b**) summary and domain scores^a^ for adult patients with PIDD compared with patients with other chronic conditions and US general population. ^a^Physical functioning, role physical, bodily pain, and general health are domains of the physical component scores; vitality, social functioning, role emotional, and mental health are domains of the mental component score. *p < 0.05 compared with PIDD sample. **p < 0.01 compared with PIDD sample. CHF, congestive heart failure; COPD, chronic obstructive pulmonary disease; IgG, immunoglobulin G; MID, minimally important difference; PIDD, primary immunodeficiency diseases; SF-36, short form health survey; US, United States

Seventeen of the 21 adults enrolled completed SF-36v2 assessments prior to IVIG treatment initiation (baseline); the 3 adult patients who did not complete the assessments after 12 months of treatment were excluded from the baseline SF-36v2 analyses. Prior to treatment initiation, adult patients who were diagnosed with PIDD had diminished HRQOL compared with the general US population and patients with other chronic conditions (Fig. 1a and b). The mean Physical Component Summary (PCS) score for adult patients (*N* = 17) with PIDD was 37.4, which was significantly lower than in the general US population (50.5; *N* = 4,024; *p* < 0.01), and in patients with various types of cancer, excluding skin cancer (44.4, *N* = 311; *p* < 0.05) or chronic back pain (44.1, *N* = 893; *p* < 0.05) (see Fig. 1a). In the physical health domains of the SF-36v2 (physical functioning, role—physical, bodily pain, general health), adult patients with PIDD prior to treatment had significantly lower scores for all 4 domains compared with the general US population (*p* < 0.05), and compared with patients suffering from other chronic conditions for role-physical (*p* < 0.05 for rheumatoid arthritis, cancer, chronic back pain) and general health (*p* < 0.05 for rheumatoid arthritis, COPD, and CHF; *p* < 0.01 for cancer and chronic back pain).

Similarly, the mean Mental Component Summary (MCS) score was significantly lower for adult patients with PIDD (41.6; *n* = 17) compared with the general US population (49.2, *N* = 4,024; *p* < 0.05) (see Fig. 1b). Furthermore, the MCS for adult patients with PIDD was lower, but not statistically significant, than MCS values reported by patients with other chronic conditions (including rheumatoid arthritis, COPD, CHF, cancer, and chronic back pain). For the mental health domains of the SF-36v2, adult patients with PIDD experienced significantly diminished (*p* < 0.05) vitality (38.2 vs. 49.4), social functioning (33.6 vs. 49.8), and role—emotional (41.0 vs. 49.7), but not mental health, compared with the general US population. Adult patients with PIDD also reported significantly lower scores for social functioning (*p* < 0.05 for rheumatoid arthritis, COPD, CHF, cancer, and chronic back pain) and vitality (*p* < 0.05 for CHF and cancer) relative to age-matched patients with chronic diseases.

**Fig. 2:**
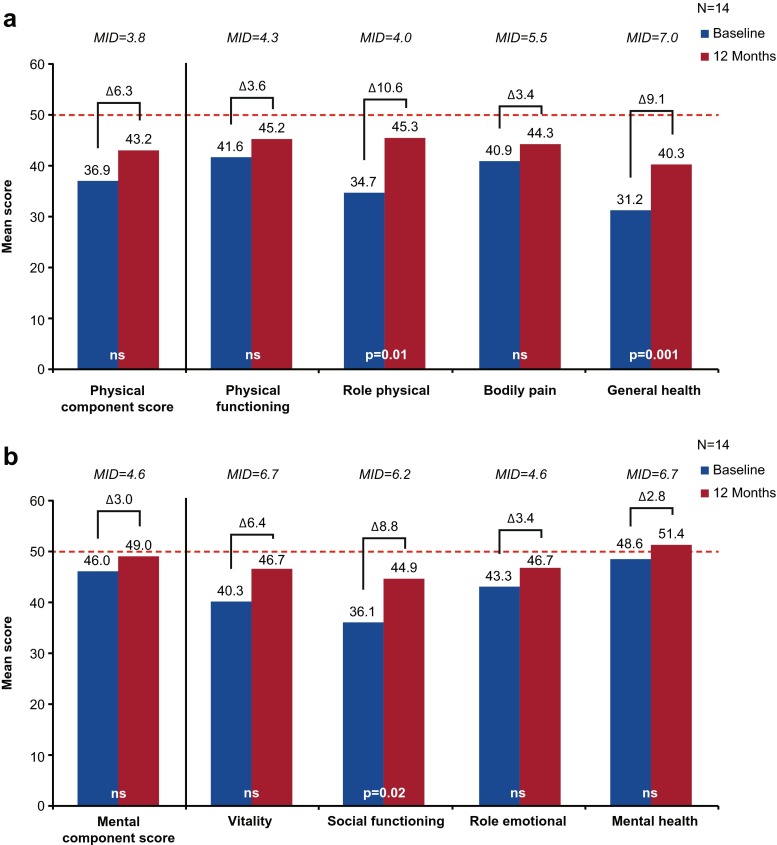
SF-36 physical (**a**) and mental (**b**) component and domain scores^a^ of adult patients with PIDD at baseline and at 12 months following IgG treatment initiation. ^a^Physical functioning, role physical, bodily pain, and general health are domains of the physical component scores; vitality, social functioning, role emotional, and mental health are domains of the mental component score. ------- SF-36 US general population norms, Δ, difference. MID, minimally important difference; ns, not significant; PIDD, primary immunodeficiency disease; SF-36, 36-item Short Form Health Survey; US, United States

Of the 17 adult patients assessed for QOL at baseline, 14 were assessed twelve months after IgG treatment initiation. Comparing baseline and follow up data for these 14 patients, increases in both PCS (from 36.9 to 43.2; Fig. 2a) and MCS (from 46.0 to 49.0; Fig. 2b) were observed. While these differences were not significant, the change in PCS (6.3) after IgG treatment exceeded the MID of 3.8 and may be clinically relevant. Furthermore, significant and clinically relevant increases were observed in a number of physical and mental individual domain scores, including role-physical (*p* = 0.01; observed change = 10.6, *MID* = 4.0), general health (*p* < 0.01; observed change = 9.1, *MID* = 7.0), and social functioning (*p* = 0.02; observed change = 8.8, *MID* = 6.2) (see Figs. 2a and b).

### QOL Assessment in Pediatric Patients

**Fig. 3:**
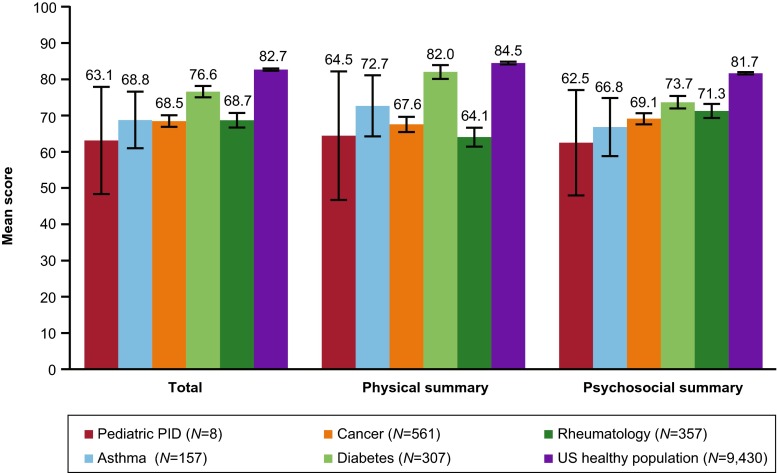
PedsQL total, physical, and psychosocial summary scores of pediatric patients with PIDD compared with pediatric patients with other chronic conditions and the US healthy population. The black bars represent the 95 % confidence intervals. PedsQL, pediatric quality of life; PIDD, primary immunodeficiency disease; US, United States

Eight of the 10 enrolled pediatric patients completed PedsQL assessments at baseline; the 2 patients who did not complete both assessments were excluded from the PedsQL analyses. According to baseline PedsQL scores, pediatric patients diagnosed with PIDD experienced diminished HRQOL compared with the healthy pediatric population (Fig. 3). Those with PIDD (*N* = 8) had significantly lower (*p* = 0.05) PedsQL total scores (63.1 [95 % confidence interval; CI: 48.4–77.9] vs. 82.7 [95 % CI: 82.4–83.0]), physical summary scores (64.5 [95 % CI: 46.7–82.2] vs. 84.5 [95 % CI: 84.1–84.9]), and psychosocial summary scores (62.5 [95 % CI: 48.0–77.0] vs. 81.7 [95 % CI: 81.3–82.0]) compared with the general US population (*N* = 9,430). The total scores, physical summary scores, and psychosocial summary scores were also lower for pediatric patients with PIDD relative to those with asthma (*N* = 157), diabetes (*N* = 307), cancer (*N* = 561), and rheumatology conditions (*N* = 357), with the exception of physical summary score comparability of PIDD with rheumatology conditions.

**Fig. 4:**
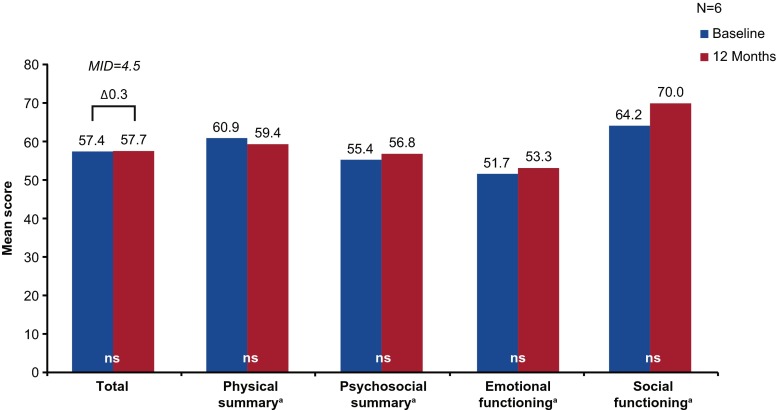
PedsQL scores among pediatric patients with PIDD at baseline and at 12 months following IgG treatment initiation. ^a^MIDs were not available for summary and component scores. IgG, immunoglobulin G; MID, minimally important difference; ns, not significant; PedsQL, Pediatric Quality of Life; PIDD, primary immunodeficiency diseases; US, United States

Of the 8 pediatric patients with PIDD assessed at baseline, 6 completed assessments 12 months following IgG treatment initiation. No significant or clinically meaningful differences in total, physical summary, or psychosocial summary scores were reported (Fig. 4).

**Fig. 5:**
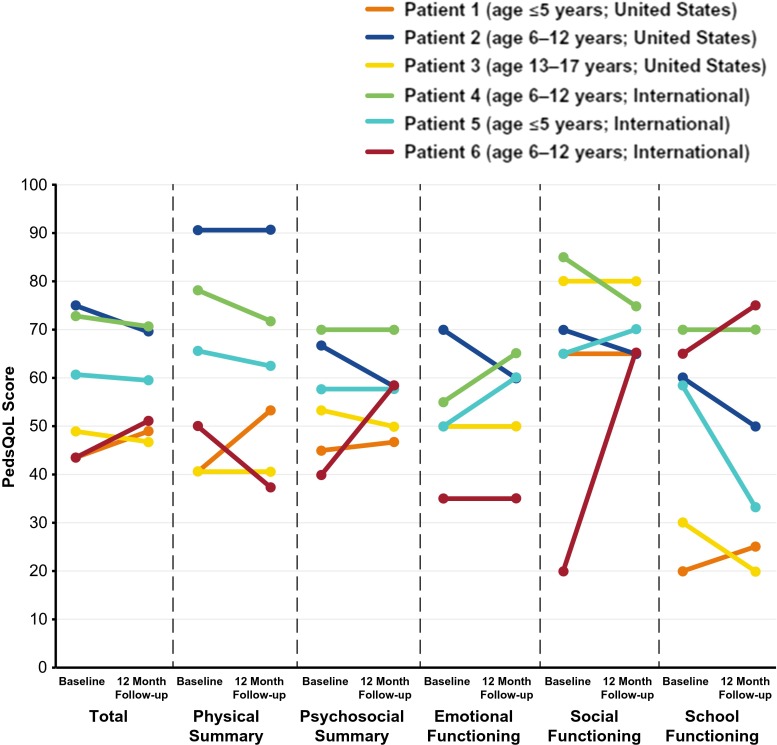
PedsQL scores among individual pediatric patients with PIDD at baseline and at 12 months following IgG treatment initiation. IgG, immunoglobulin G; PedsQL, Pediatric Quality of Life; PIDD, primary immunodeficiency diseases

Figure 5 illustrates the change in PedsQL scores from baseline to 12 months for each individual pediatric patient. Examination of these individual changes shows a wide variation in the PedsQL scores between patients.

### Health Resource Utilization in Adult and Pediatric Patients

Health resource utilization among patients with PIDD prior to and after IgG treatment initiation is summarized in Table 2. Adult and pediatric patients as a group reported a significantly lower mean number of hospital admissions (*n* = 25; 1.8 vs 0.21; *p* < 0.01), serious infections (*n* = 18; 10.9 vs 3.3; *p* < 0.01), and antibiotic prescriptions (*n* = 22; 7.1 vs 3.0; *p* < 0.01) after initiation of IgG treatment compared with baseline. An adult-only sub analysis further showed a significant decrease in mean emergency room visits associated with IgG treatment (*n* = 17; 1.8 at baseline vs 0.4 post-treatment initiation, *p* = 0.04). Additionally, pediatric patients had significantly fewer mean number of unscheduled office visits (*n* = 7; 20.0 vs 8.3, *p* = 0.01) after treatment initiation compared with baseline.

**Table 2:** Mean health resource utilization per patient before and 12 months after IgG treatment initiation

Health Resource Utilization	Adult Patients (Age ≥18 Years)				Pediatric Patients (Age <18 Years)			
	n	Baseline (12-Months Pre-treatment),^a^ Mean (SD)	12- Months, Mean (SD)	p-value	n	Baseline (12-Months Pre-treatment),^a^ Mean (SD)	12 Months, Mean (SD)	p-value
Regularly scheduled office visits	16	7.1 (7.8)	8.9 (8.7)	NS	7	10.6 (5.3)	11.3 (5.7)	NS
Unscheduled office visits	17	11.6 (15.6)	3.8 (5.4)	NS	7	20.0 (9.2)	8.3 (6.8)	0.01
Emergency room visits	17	1.8 (2.4)	0.4 (0.7)	<0.05	7	6.3 (8.3)	1.9 (3.3)	NS
Hospital admissions	17	1.4 (2.4)	0.2 (0.4)	0.05	8	2.8 (2.1)	0.4 (0.5)	0.01
Length of stay^b^	--	--	--		3	46.0 (46.1)	7.7 (8.1)	NS
Number of serious infections^c^	12	8.0 (7.2)	2.5 (1.6)	<0.05	6	16.7 (9.0)	5.0 (2.1)	<0.05
Days missed from work or school^d^	12	25.5 (36.9)	37.4 (103.4)	NS	4	63.5 (77.8)	46.0 (36.5)	NS
Number of antibiotic prescriptions	16	5.5 (4.0)	2.5 (2.3)	<0.01	6	11.3 (9.0)	4.2 (2.6)	NS

^a^Pre-enrollment data was annualized

^b^Only 1 adult patient had length-of-stay data

^c^Serious infections defined as: acute or chronic sinusitis, bronchitis, pneumonia, ear infection, or other acute or chronic infection

^d^Four adult patients who were retired were excluded from this analysis

*ER* emergency room, *IgG* immunoglobulin G, *NS* not significant, *SD* standard deviation

### Patient Productivity

Adult and pediatric patients with PIDD experienced a reduction in productivity in the 6 months preceding treatment initiation. After initiation of IgG treatment, adult patients (*n* = 12) reported a non-significant increase in days missed from work/school (mean 25.5 days prior to IgG treatment and 37.4 days after 12 months of IgG treatment), while pediatric patients (*n* = 4) reported 63.5 and 46.0 days missed from school, prior to and after IgG treatment initiation, respectively (see Table 2).

### QOL Assessment in Caregivers of Pediatric Patients

**Fig. 6:**
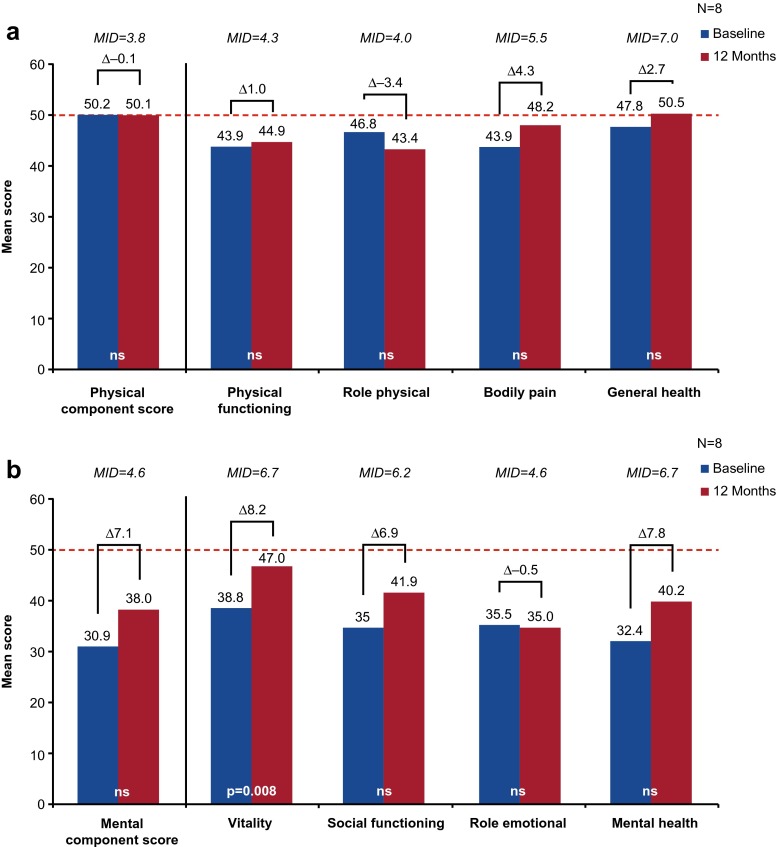
SF-36 physical (**a**) and mental (**b**) component and domain scores^a^ for caregivers of pediatric patients with PIDD at baseline and at 12 months following IgG treatment initiation. ^a^Physical functioning, role physical, bodily pain, and general health are domains of the physical component scores; vitality, social functioning, role emotional, and mental health are domains of the mental component score. ------- SF-36 US general population norms. Δ, difference. MID, minimally important difference; ns, not significant; PIDD, primary immunodeficiency disease; SF-36, 36-item Short Form Health Survey; US, United States

Eight caregivers completed SF-36v2 assessments at baseline and 12 months following IgG treatment initiation; the 2 caregivers who did not complete both assessments were excluded from the SF-36 analyses. While caregivers did not report a significant difference in PCS (Fig. 6a) or MCS (Fig. 6b) following IgG treatment, MCS was numerically higher (38.0vs 30.9) and clinically relevant (observed change = 7.1; *MID* = 4.6) after 12 months of treatment compared with baseline. Furthermore, compared with baseline, caregivers also reported a significantly higher (47.0 vs. 38.8, *p* < 0.01) and clinically relevant (observed change = 8.2, *MID* = 6.7) domain score for vitality after treatment (see Fig. 6b). Clinically relevant (but not statistically significant) increases in social functioning (observed change = 6.9, *MID* = 6.2) and mental health (observed change = 7.8, *MID* = 6.7) were also reported (see Fig. 6b).

## Discussion

Recent data suggest that although the prevalence of PIDD is higher than once thought [41], patients continue to experience significant delays in diagnosis [17, 25, 26]. The present study sought to gain insight into the burden of disease among newly diagnosed patients with antibody deficiencies and to explore HRQOL and HRU following the initiation of IgG treatment. The SF-36 and PedsQL surveys used to assess HRQOL in adult and pediatric patients, respectively, in the current survey have previously been used in other studies of patients with PIDD [18, 21, 22, 28–30, 34]. The results presented herein suggest that adult and pediatric patients with PIDD have diminished HRQOL and productivity prior to the initiation of IgG treatment, and that following 12 months of IgG, adult (but not pediatric) patients experienced improved HRQOL, as reflected by significant and clinically relevant increases in 2 physical health and 1 mental health domain scores in the SF-36 survey. Furthermore, the data also suggest improved HRQOL for pediatric patients’ caregivers, who reported a significant increase in their own vitality following 12 months of IgG treatment of their children. Notably, clinically meaningful increases in social functioning and mental health domain scores were also observed for caregivers. In addition, significant decreases were reported in a number of HRU categories for both adult and pediatric patients. Compared with baseline, however, the number of missed days at work (adults) or school (pediatric patients) was not significantly different following IgG treatment.

The wide variation in the PedsQL scores observed between individual pediatric patients (see Fig. 5) suggests that while some pediatric patients reported an improvement after 12 months of treatment, others reported no change or worsening of their HRQOL. However, these data are difficult to interpret due to the low number of pediatric patients in our study population. Although the wide variation may reflect a true variation within the pediatric PIDD population, the disparity may also reflect an aberration within our small study sample; specific trends may be elucidated with a larger sample size.

While several studies have investigated HRQOL in adult and pediatric patients with PIDD following IgG replacement [18, 21–23, 28–32], none have compared HRQOL in the same cohort prior to and after treatment initiation. In general, data from previous studies have shown a lower HRQOL in patients with PIDD compared with the general population, including adult patients with CVID [18] and select IgA deficiency [28]. Similar results were reported from studies focusing on pediatric patients in which significantly lower PedsQL scores were observed in those with PIDD compared with healthy individuals [29, 31, 32]. However, in a survey of 25 adult males with XLA, numerically, but not significantly, lower QOL scores, compared with the general US population, were reported [23].

In the current study, QOL and HRU were assessed within the same patient cohort both prior to PIDD diagnosis and following 12 months of IgG replacement therapy. At baseline, adults with PIDD had significantly lower PCS and MCS (SF-36) compared with the general US population and significantly lower PCS compared with patients with chronic back pain and cancer. The mental burden, on the other hand, was similar between adults with PIDD and patients with other chronic conditions. Similarly, pediatric patients diagnosed with PIDD experienced a substantial HRQOL burden of disease, as was evident from the lower PedsQL scores than the general US population. When compared with pediatric patients with other chronic diseases, including cancer, asthma, and rheumatologic diseases, scores were either similar or lower.

Patients were followed after 12 months of IgG treatment. After treatment, patients documented lower HRU with regards to hospital admissions, serious infections, and antibiotic prescriptions. Adult patients reported a significant increase in the role-physical and general health domain scores of PCS and in the vitality domain of the MCS. These results are consistent with other studies that have noted an inverse relationship between persistence of symptoms (ie, serious infections, chronic diarrhea) and SF-36 scores [18, 30]. While previous studies have primarily indicated a QOL deficit in patients with PIDD who are already receiving treatment compared with healthy populations or patients with other chronic diseases, the current study indicates that adult patients do experience an improvement in QOL following IgG treatment initiation. However, this study did not collect interim assessment data (ie, within the 12 months of treatment); therefore, an opportunity was missed to compare differences in improvement during, eg, the first 6 months of IgG treatment to the last 6 months of IgG treatment.

In addition to evaluating patients with PIDD, the current study also surveyed caregivers (mothers) of pediatric patients with PIDD. Notably, caregivers reported a significant increase in the vitality domain as well as clinically relevant differences in the social functioning and mental health domains of MCS after their children initiated IgG replacement, suggesting that the QOL benefit may extend beyond the patients themselves. However, both pediatric and caregiver results should be interpreted with caution due to the particularly low number of subjects in these analyses (as further illustrated by the variation in individual pediatric PedsQL data in Fig. 5).

The present study focused on patients with antibody deficiencies, and the small sample size (particularly the low number of pediatric patients and their caregivers) presented here may not accurately reflect the general population of patients with PIDD. Further, since a power calculation was not conducted, this study may have been underpowered to adequately detect the true effect of treatment on patient HRQOL. Because of the small number of patients in the current study, additional analyses are limited. For example, it is not statistically plausible to compare the socioeconomic status and geographical areas of the patients. It is also important to consider that self-reported survey results are always subject to potential bias because patients and caregivers may not accurately remember or report details of their condition. In the current analysis, we compared scores from patients with PIDD (prior to and after treatment with IgG) to the SF-36v2 normative database without information regarding the treatment status of patients with chronic conditions included in the database. If some or all of the patients with chronic conditions had received treatments, it might impact their HRQOL and subsequently influence our comparison. Furthermore, while prior research has shown that using the US-based SF-36v2 norms for interpreting scores across adult respondents from different countries is appropriate (between-score correlations of 0.98–1.00 for physical and mental components in all 9 European countries evaluated) [39], the comparability with the patients from our sample from other countries may be limited because the SF-36v2 normative database contains only US respondents. Similarly, the PedsQL survey has been translated (with cultural adaptations) and validated for use in numerous countries (PedsQL: http://pedsql.org/pedsql2.html). Although the PedsQL has been validated among different ethnic and racial backgrounds within a US population [38], to our knowledge no cross-country validation studies of the PedsQL survey have been published.

## Conclusions

In the current analysis, prior to IgG replacement, adult and pediatric patients with PIDD suffered from diminished HRQOL compared with the general US population and patients with other chronic diseases. In addition, patients experienced diminished productivity due to the number of work and/or school days that were missed because of their condition. Twelve months after IgG treatment initiation, adult patients and caregivers of pediatric patients reported an improvement in QOL. Additionally, HRU decreased for the overall population, including pediatric patients.
